# The Effect of Insomnia on Cortical Excitability in Patients With Generalized Anxiety Disorder

**DOI:** 10.3389/fpsyt.2018.00755

**Published:** 2019-01-10

**Authors:** Zhaoyang Huang, Shuqin Zhan, Chao Chen, Ning Li, Yan Ding, Yue Hou, Li Wang, Yuping Wang

**Affiliations:** ^1^Department of Neurology, Xuanwu Hospital, Capital Medical University, Beijing, China; ^2^Beijing Key Laboratory of Neuromodulation, Beijing, China; ^3^Key Laboratory of Complex System Control Theory and Application, Tianjin University of Technology, Tianjin, China

**Keywords:** insomnia, generalized anxiety disorder, somatosensory evoked potential, recovery function, right parietal cortex

## Abstract

The high rate of comorbidity between insomnia and anxiety disorders have been confirmed by previous studies. However, the underlying neurobiological correlates of the relationship between insomnia and anxiety disorders are largely unknown. The aim of the present study was to investigate the effect of insomnia on cortical excitability in patients with generalized anxiety disorder (GAD) by examining the recovery functions of median nerve somatosensory evoked potentials (SEPs) in patients with GAD without insomnia and patients with GAD comorbid with insomnia. We studied the recovery functions of median nerve SEPs in 12 medication-naive patients with GAD without insomnia, 15 medication-naive patients with GAD comorbid with insomnia, and 15 age and sex matched healthy controls. SEPs in response to single stimulus and paired stimuli at interstimulus intervals (ISIs) of 20, 60, 100, and 150 ms were recorded. The recovery function of the P25 component showed significantly reduced suppression in patients with GAD without insomnia as compared to patients with GAD comorbid with insomnia and healthy controls. There were no significant differences in the recovery functions of median nerve SEPs between patients with GAD comorbid with insomnia and healthy controls. The present study suggested that the cortical excitability of right parietal cortex increased in patients with GAD without insomnia, and cortical excitability in patients with GAD comorbid with insomnia was modulated by insomnia. Our findings provide new insights into the underlying neurobiological correlates of the effects of insomnia on GAD, which could ultimately be used to inform clinical intervention.

## Introduction

Epidemiological studies have shown a high rate of comorbidity between insomnia and anxiety disorders. A number of longitudinal studies indicated that the relationship between insomnia and anxiety disorders is bidirectional. Insomnia contributes to the development of anxiety disorders, and anxiety disorders result in insomnia (1, 2). Treating chronic insomnia can often reduce the severity of anxiety symptoms, and similarly, treating anxiety can often improve insomnia (3). Generalized anxiety disorder (GAD) is the most common anxiety disorder, which is characterized by pervasive worry, difficulty concentrating, feeling restless, easily fatigued, muscle tension, and sleep disturbances (4). About 75% of patients with GAD have insomnia (5, 6). Sleep difficulties are included in the diagnostic criteria for GAD (4). Previous studies showed that GAD independently predicted insomnia, and higher levels of insomnia significantly predicted higher levels of GAD (7). Despite the well-documented association between insomnia and anxiety, the underlying neurobiological correlates remain unclear.

Patients with anxiety disorders characteristically show physical and psychological arousal (8). It is believed that anxiety is associated with alterations in brain excitability (9). Previous studies found that patients with anxiety disorders had significant high right parietal activity (10–13). These results suggested that the cortical excitability of right parietal lobe might be abnormal in patients with anxiety disorders. Previous studies also found that the functions of right parietal lobe in patients with GAD are abnormal. Etkin et al. (14) found an altered functional connectivity between bilateral posterior parietal cortex and the amygdala in patients with GAD using fMRI. Wu et al. (15) noted that patients with GAD had more activity in their right parietal lobes during vigilant tasks. Brambilla et al. (16) found that white-matter connectivity is impaired in the right parietal lobe in patients with GAD. These findings indicated that the cortical excitability of right parietal lobe might be abnormal in patients with GAD.

Several previous studies have revealed abnormal cortical excitability in anxiety disorders, including obsessive-compulsive disorder (17), social anxiety disorder (18), post-traumatic stress disorder (19), and generalized anxiety disorder (20). So far, there have been only one published study investigating cortical excitability in patients with GAD. Li CT et al. measured motor cortical excitability of patients with GAD using paired-pulse transcranial magnetic stimulation (20). They found that GAD patients had significantly lower intracortical facilitation (ICF). They concluded that GAD was associated with impaired intracortical facilitation, and such ICF deficits predicted the severity of anxiety. However, they did not consider the impact of insomnia on cortical excitability in patients with GAD.

Previous studies demonstrated that insomnia have a significant impact on GAD. Insomnia and sleep deprivation can significantly increase anxiety (21), and GAD patients with higher levels of insomnia have higher levels of anxiety symptoms (7). Thus, we assumed that insomnia could lead to changes in cortical excitability of patients with GAD, and cortical excitability might be different between patients with GAD without insomnia and patients with GAD comorbid with insomnia.

Paired-pulse stimulation techniques are used as common tools to investigate cortical excitability and cortical plastic changes. The recovery function of cortical somatosensory evoked potential (SEPs) component in the paired-pulse paradigm, which has been used in our previous study (22), has been applied to study the cortical excitability in patients with various psychiatric and neurological disorders (23, 24). When paired stimuli are delivered at different inter-stimulus intervals (ISIs), the amplitude of the SEP evoked by the second stimulus is suppressed depending on the interstimulus interval. The longer is the ISI, the higher is the amplitude of the SEP evoked by the second stimulus, until a complete amplitude recovery is observed (25–27). To improve the understanding of the neurophysiological effects of insomnia on GAD, the present study investigated alterations of cortical excitability of right parietal cortex in patients with GAD without insomnia and patients with GAD comorbid with insomnia by examining the recovery functions of median nerve SEPs. We hypothesized that the cortical excitability of right parietal cortex might be abnormal in patients with GAD without insomnia, and the cortical excitability of right parietal cortex in patients with GAD comorbid with insomnia might be modulated by insomnia.

## Methods

### Participants

We studied the recovery functions of median nerve SEPs in 12 medication-naive patients with GAD without insomnia, 15 medication-naive patients with GAD comorbid with insomnia, and 15 age and sex matched healthy controls. All participants were recruited from neurology outpatient clinics of Xuanwu Hospital. All procedures of this study were approved by the Institutional Review Board of Xuanwu Hospital and written informed consent was obtained from each participant.

All participants were interviewed and examined by two experienced neurologists. The interview included the administration of the Pittsburgh Sleep Quality Index (PSQI) (28), the Hamilton Anxiety Rating Scale (HAMA) (29) and the Hamilton Depression Rating Scale (HAMD, 24-item version) (30).

All participants aged from 18 to 60 years old. All patients met diagnostic criteria for GAD according to the Structured Clinical Interview for the Diagnostic and Statistical Manual of Mental Disorders-IV-TR (DSM-IV). Patients with GAD comorbid with insomnia also met diagnostic criteria for insomnia based on criteria for insomnia related to another mental disorder from the DSM-IV with duration of insomnia ≥ 3 months. Patients with GAD without insomnia were required to have the HAMA score of ≥ 14, the PSQI score of < 7, and the HAMD-24 score of < 20. Patients with GAD comorbid with insomnia were required to have the HAMA score of ≥ 14, the PSQI score of ≥ 7, and the HAMD-24 score of < 20. All patients were required to have no prior history of other psychiatric diseases, including all types of anxiety disorders other than GAD, depression, substance or alcohol abuse or dependence, and other sleep disorders.

Healthy controls were required to have no history of psychiatric diseases and sleep disorders, and have the PSQI score of < 7, the HAMA score of < 7, the HAMD-24 score of < 8.

Exclusion criteria for both groups were as follows: evidence of neurological or other physical diseases such as respiratory, cardiac, renal, hepatic, and endocrinal diseases as assessed by clinical history, physical examination or routine laboratory tests; any medication that might affect central nervous system within 14 days; irregular sleep patterns associated with shift work, frequent travel or personal preference (as indicated by a weekly variation > 3 h in bedtime or wake time, or time in bed duration < 5.5 or > 10 h per night); concurrent psychotherapy or counseling; pregnancy or breastfeeding women.

### SEPs Recording Procedure

For SEPs recording, we used the same method and parameters as in our earlier study (22). Left median nerve was stimulated at the wrist at an intensity fixed at about 1.2 times the motor threshold (stimulus duration: 0.2 ms, stimulus rate: 1 Hz). SEPs were recorded (the Neuropack M1 MEB-9200 EP/EMG measuring system, Nihon Kohden Corporation, Japan) with the recording electrodes placed over the ipsilateral Erb point, the spinous process of the sixth cervical vertebra (Cv6), and the contralateral parietal area (C4', 2 cm posterior to the C4 placement of the international 10–20 system). All recording electrodes were referred to the right earlobe.

We recorded the N9 potential from the ipsilateral Erb's point, the N13 potential from Cv6, and the P14, N20, and P25 potentials from the parietal region contralateral to the stimulation side.

### SEPs Recovery Functions

Recovery functions of SEPs were studied using the same method and parameters as in our earlier study (22). Paired stimuli of equal intensity were given at ISIs of 20, 60, 100, and 150 ms. Single trial SEPs were taken as control. The sequences of these trials were randomized among the subjects. At least three hundred sweeps were averaged for each condition. To ascertain reproducibility of results, SEPs of each condition (single stimulus, and paired stimuli at ISIs of 20, 60, 100, and 150 ms) were recorded at least twice, one trial after another trial. Then we obtained the average SEPs time series of each condition used for subtraction. To obtain SEPs evoked by the test stimulus (T-SEPs), we subtracted SEPs evoked by single stimulus alone (S-SEPs) from those elicited with paired stimuli.

We measured amplitudes of SEPs from the preceding peak (peak-to-peak) to prevent the impact of baseline shift on the results. For SEPs recovery functions, the amplitudes of each component in the subtracted SEPs waveform were measured. Then we calculated the relative amplitude ratios of T-SEPs to those of the corresponding S-SEPs at different ISIs. Finally, we obtained SEP recovery curves (SEP-Rs) by plotting the amplitude ratios of T-SEP/S-SEP against the interstimulus intervals. The value of ratio ≥ 1 means that there is no suppression.

### Statistical Analysis

All statistical analysis was carried out with SPSS version 19.0 for Windows (SPSS Inc., Chicago, IL). The demographic and clinical characteristics at baseline were compared among the three groups using Pearson's chi-square test and One-way Analysis of Variance (ANOVA) followed by the LSD *post-hoc* test. For the amplitudes of SEPs obtained by single stimulus, we used one-way ANOVA test. For recovery functions obtained by paired stimuli, we employed a repeated measures ANOVA with ISI as the within-subjects factor and group as the between-subjects factor. A *p* ≤ 0.05 was considered statistically significant results.

## Results

### Demographic and Clinical Characteristics

The demographic and clinical characteristics of the participants, including age, sex, and the scores of PSQI, HAMA, and HAMD, are summarized in Table 1.

**Table 1:** Demographic and clinical characteristics of the participants.

**Variable**	**Controls**	**GAD without insomnia**	**GAD comorbid with insomnia**
	**Mean (*SD*)**	**Mean (*SD*)**	**Mean (*SD*)**
Cases	15	12	15
Male/Female	6/9	4/8	6/9
Age	43.27 (10.31)	40.08 (10.92)	39.87 (8.65)
PSQI	2.20 (1.08)	3.33 (1.07)	14.47 (3.54)^b^^,^^c^
HAMA	3.60 (2.44)	16.75 (2.30)^a^	20.13 (3.89)^b^^,^^d^
HAMD	5.00 (1.31)	9.50 (2.39)^a^	13.67 (2.50)^b^^,^^c^

*PSQI, Pittsburgh Sleep Quality Index; HAMA, Hamilton Anxiety Rating Scale; HAMD, Hamilton Depression Rating Scale*.

a *Indicates significant differences between patients with GAD without insomnia and the controls (p < 0.01)*.

b *Indicates significant differences between patients with GAD comorbid with insomnia and the controls (p < 0.01)*.

c *Indicates significant differences between patients with GAD comorbid with insomnia and patients with GAD without insomnia (p < 0.01)*.

d *Indicates significant differences between patients with GAD comorbid with insomnia and patients with GAD without insomnia (p < 0.05)*.

There were no significant differences among the three groups with respect to age (*F* = 0.54, *p* = 0.59) and sex (χ^2^ = 0.16, *p* = 0.92). The PSQI score was significantly higher in patients with GAD comorbid with insomnia than the other two groups (*p* < 0.01). There were no significant differences in PSQI scores between patients with GAD without insomnia and the controls (*p* > 0.05). The HAMA scores were significantly higher in patients with GAD comorbid with insomnia (*p* < 0.01) and patients with GAD without insomnia (*p* < 0.01) than the controls. The HAMA scores were significantly higher in patients with GAD comorbid with insomnia than patients with GAD without insomnia (*p* < 0.05). Similarly, the HAMD scores were significantly higher in patients with GAD comorbid with insomnia (*p* < 0.01) and patients with GAD without insomnia (*p* < 0.01) than the controls. The HAMD scores were significantly higher in patients with GAD comorbid with insomnia than patients with GAD without insomnia (*p* < 0.01).

### Single-Pulse SEPs

Mean values and standard deviations of the amplitudes of SEPs components in the single-pulse condition are shown in Table 2.

**Table 2:** Mean amplitudes (μV) of SEPs components in the single stimulus condition.

**Components**	**Controls**	**GAD without insomnia**	**GAD comorbid with insomnia**	***p*-value**
N9	6.88 (3.03)	6.06 (1.76)	6.95 (2.40)	0.14
N13	3.47 (0.77)	3.19 (0.57)	3.42 (0.77)	0.10
N20	2.99 (0.94)	3.29 (1.34)	3.08 (0.98)	0.36
P25	4.78 (2.28)	5.58 (2.95)	5.10 (2.79)	0.30

*Each value is expressed as mean (standard deviation)*.

In the single stimulus condition, there were no significant differences in the amplitudes of SEPs components among the three groups (*p* > 0.05).

### SEPs Recovery Functions

Mean values and standard deviations of the amplitude ratios of T-SEP/S-SEP at different ISIs are shown in Table 3.

**Table 3:** The amplitude ratios of T-SEP/S-SEP at different ISIs in the three groups.

**ISIs**		**N9**	**N13**	**N20**	**P25**
20 ms	Controls	0.92 (0.25)	0.84 (0.24)	0.88 (0.29)	0.72 (0.34)
	GAD without insomnia	0.94 (0.27)	0.88 (0.14)	0.78 (0.31)	1.23 (0.33)
	GAD comorbid with insomnia	1.04 (0.30)	0.78 (0.18)	0.79 (0.19)	0.47 (0.29)
60 ms	Controls	1.03 (0.30)	0.84 (0.21)	0.84 (0.28)	0.55 (0.21)
	GAD without insomnia	1.07 (0.55)	0.91 (0.27)	0.93 (0.36)	0.80 (0.29)
	GAD comorbid with insomnia	0.87 (0.15)	0.82 (0.23)	0.85 (0.21)	0.64 (0.26)
100 ms	Controls	1.04 (0.27)	0.96 (0.19)	0.89 (0.35)	0.73 (0.16)
	GAD without insomnia	1.10 (0.15)	0.98 (0.24)	0.86 (0.26)	0.88 (0.12)
	GAD comorbid with insomnia	1.09 (0.31)	0.96 (0.18)	0.92 (0.11)	0.86 (0.28)
150 ms	Controls	1.07 (0.32)	0.96 (0.13)	0.93 (0.17)	0.82 (0.19)
	GAD without insomnia	1.11 (0.35)	1.05 (0.28)	1.11 (0.24)	0.79 (0.24)
	GAD comorbid with insomnia	0.96 (0.12)	0.95 (0.12)	0.94 (0.18)	0.81 (0.13)

*ISIs, Interstimulus intervals. Each value is expressed as mean (standard deviation)*.

The N9 component evoked by the test stimulus in healthy controls were suppressed (the amplitude ratios of T-SEP/S-SEP ratios < 1.0) at ISI of 20 ms, and recovered at ISI of 60 ms. In patients with GAD without insomnia and patients with GAD comorbid with insomnia, the N9 component behaved similarly to the normal controls. The repeated measures ANOVA showed that there were no significant differences among the three groups (*F* = 0.80, *p* = 0.46).

The N13 component evoked by the test stimulus in healthy controls were suppressed at all ISIs of 20, 60, 100, and 150 ms. In patients with GAD without insomnia and patients with GAD comorbid with insomnia, the N13 component behaved similarly to the normal controls. The repeated measures ANOVA showed that there were no significant differences among the three groups (*F* = 2.35, *p* = 0.11).

The N20 component evoked by the test stimulus in healthy controls were suppressed at all ISIs of 20, 60, 100, and 150 ms. In patients with GAD without insomnia, the N20 component evoked by the test stimulus recovered at ISI of 150 ms. In patients with GAD comorbid with insomnia, the N20 component behaved similarly to the controls. The repeated measures ANOVA showed that there were no significant differences among the three groups (*F* = 0.31, *p* = 0.74).

The P25 component evoked by the test stimulus in healthy controls were suppressed at all ISIs of 20, 60, 100, and 150 ms. In patients with GAD without insomnia, the P25 component evoked by the test stimulus were not suppressed at ISI of 20 ms, and suppressed at ISIs of 60, 100, and 150 ms. In patients with GAD comorbid with insomnia, the P25 component evoked by the test stimulus were suppressed at all ISIs. The repeated measures ANOVA showed that the recovery functions of the P25 component were significantly different among the three groups (*F* = 10.96, *p* < 0.01). *Post-hoc* tests showed significant differences between the patients with GAD without insomnia and the controls (*p* < 0.01), the patients with GAD without insomnia and the patients with GAD comorbid with insomnia (*p* < 0.01), but no significant differences between the patients with GAD comorbid with insomnia and the controls (*p* = 0.89).

**Figure 1:**
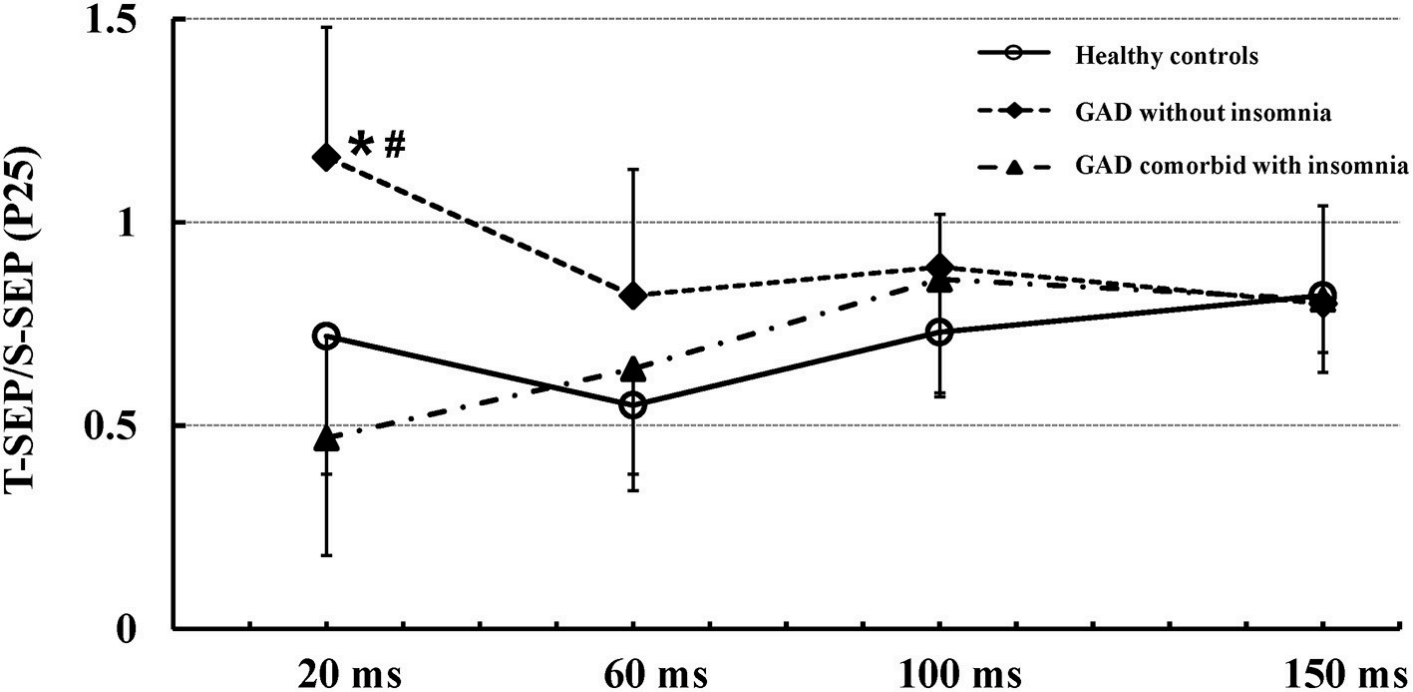
Mean (± SD) recovery curves of the P25 component in the three groups. The recovery function of the P25 component showed significantly reduced suppression in patients with GAD without insomnia (solid diamonds, dashed line) as compared to patients with GAD comorbid with insomnia (solid triangles, dash-dotted line) and healthy controls (open circles, solid line). There were no significant differences in the recovery function of the P25 component between patients with GAD comorbid with insomnia and healthy controls. *Indicates significant difference between patients with GAD without insomnia and healthy controls (*p* < 0.01). ^#^Indicates significant difference between patients with GAD without insomnia and patients with GAD comorbid with insomnia (*p* < 0.01).

Figure 1 shows mean (± SD) recovery curves of the P25 component in the three groups.

## Discussion

In the present study, we investigated changes in cortical excitability in patients with GAD without insomnia and patients with GAD comorbid with insomnia by examining the recovery functions of median nerve SEPs. Our findings demonstrated that patients with GAD without insomnia exhibited reduced suppression of the cortical P25 component, but patients with GAD comorbid with insomnia showed no significant differences in the recovery functions of median nerve SEPs compared with the other two groups.

Previous studies suggested that the N20 component originates mainly in Brodmann's area 3b, and the P25 potential is generated by neurons in the Brodmann Areas 1and 2 of the parietal cortex (31, 32). It has been proposed that the N20 component reflects thalamocortical input to the primary somatosensory cortex, whereas the P25 component represents intracortical processing (33). The SEP recovery function of the cortical components is believed to reflect cortical excitability (24). Therefore, the normal recovery pattern of the N20 component and the disinhibited recovery pattern of the P25 component in patients with GAD without insomnia suggested an increased excitability of the parietal cortex.

The gamma-aminobutyric acid (GABA) system is believed to play a key role in the pathophysiology of GAD (34–36). Benzodiazepines, which act by enhancing inhibitory activity in the GABAergic receptor complex, are considered to be one of the most effective agents for GAD (37). Previous findings suggested that a GABA receptor-mediated mechanism in cerebral cortex might play a crucial role in the mechanism of paired-pulse inhibition (38–40). Therefore, these findings suggested that the dysfunction of inhibitory GABAergic interneurons in cerebral cortex might contribute to the disinhibited pattern of the cortical P25 component in patients with GAD without insomnia.

The level of excitability of cortical neurons depends on the balance between the GABA-related inhibitory and glutamate-related excitatory systems (41). Glutamate neurotransmitter system has also been identified to be involved in anxiety disorders (42). Pregabalin has been shown to be effective in the treatment of GAD. It works in part by reducing the release of glutamate (43). Riluzole, a drug that reduces glutamate release and consequently increase the expression of glutamate receptors, may also be effective in the treatment of mood and anxiety disorders. Other compounds, which act on the glutamate system, have also been demonstrated to have the potential to treat GAD (44, 45). Thus, these findings suggested that the glutamate neurotransmitter system might also contribute to the disinhibited pattern of the cortical P25 component in patients with GAD without insomnia.

Interestingly, the present study showed that recovery pattern of the P25 components in patients with GAD comorbid with insomnia was not significantly different from the other two groups. We proposed that the most possible mechanism is the effects of insomnia on GAD. Previous studies suggested that insomnia and sleep deprivation can significantly increase anxiety (21), and higher levels of insomnia significantly predicted higher levels of GAD (7). In the present study, the HAMA scores were significantly higher in patients with GAD comorbid with insomnia than patients with GAD without insomnia. These results suggested that insomnia aggravates the severity of the disease. Thus, the cortical excitability in patients with GAD comorbid with insomnia might be modulated by insomnia. The present study found an increased excitability of the parietal cortex in patients with GAD without insomnia. Our previous study also found that the cortical excitability of the parietal cortex increased in patients with primary insomnia (22). Previous findings suggested that a decrease in GABA and a compensatory increase in glutamate might be involved in the mechanisms of the increased excitability of the parietal cortex in both patients with GAD without insomnia and patients with primary insomnia (46, 47). We proposed that because of the impact of insomnia, there might be not enough glutamate being released, that might result in decompensation of the GABA-related inhibitory and glutamate-related excitatory systems in patients with GAD comorbid with insomnia.

Our study has several limitations. First, the present study used the recovery function of median nerve SEPs to investigate cortical excitability. This method can only reflect the regional cortical excitability of parietal lobe. Future studies could use task-related fMRI to explore differences in brain activation patterns between patients with GAD without insomnia and patients with GAD comorbid with insomnia. Then we can better understand the underlying neurobiological correlates of the relationship between insomnia and GAD. Second, we did not investigate the GABA and glutamate systems directly. Future studies could use magnetic resonance spectroscopy (MRS) to investigate differences in cortical GABAergic and glutamatergic neurotransmission between patients with GAD without insomnia and patients with GAD comorbid with insomnia. Third, the relatively small sample size is another limitation of the present study. Future studies with larger sample sizes could be conducted to confirm our conclusion.

In conclusion, the present study demonstrated that the cortical excitability of right parietal cortex increased in patients with GAD without insomnia. The cortical excitability in patients with GAD comorbid with insomnia was modulated by insomnia. The cortical GABA-related inhibitory and glutamate-related excitatory systems might play key roles in the mechanisms of the effects of insomnia on GAD. Our findings provide new insights into the underlying neurobiological correlates of the effects of insomnia on GAD, which could ultimately be used to inform clinical intervention.

## Author Contributions

YW designed the research, supervised the project and revised the article. ZH and CC performed the research, drafted the article and analyzed data. YH, NL, YD, and LW collected data and interpreted the data. SZ interpreted data and revised the draft. All authors reviewed the paper and approved it to submit.

### Conflict of Interest Statement

The authors declare that the research was conducted in the absence of any commercial or financial relationships that could be construed as a potential conflict of interest.
